# Human milk oligosaccharide profiles remain unaffected by maternal pre-pregnancy body mass index in an observational study

**DOI:** 10.3389/fnut.2024.1455251

**Published:** 2024-10-16

**Authors:** Julie Astono, Yu-Ping Huang, Ulrik Kræmer Sundekilde, Daniela Barile

**Affiliations:** ^1^Department of Food Science, Aarhus University, Aarhus, Denmark; ^2^Department of Food Science and Technology, University of California, Davis, Davis, CA, United States

**Keywords:** human milk oligosaccharides, obesity, infant growth, BMI, infant nutrition

## Abstract

Human milk oligosaccharides (HMOs) are important carbohydrates in human milk that infants cannot digest, acting as prebiotics linked to infant health. The risk of childhood obesity increases with maternal obesity, potentially mediated through the gut microbiota affected by the available HMOs. Studies on whether maternal obesity affects HMO abundance, yield conflicting results. This study aimed to investigate the HMO profile and its association with maternal obesity measured by pre-pregnancy body mass index (BMI) and infant anthropometrics. The results were discussed in the context of existing literature. 90 human milk samples were collected at 3 months postpartum from mothers in three BMI-groups: 32 normal weight (BMI: 18.5–24.99 kg/m^2^), 34 overweight (BMI: 25–30 kg/m^2^), and 24 obese (BMI > 30 kg/m^2^). The samples were analyzed using nano liquid chromatography chip quadrupole time-of-flight mass spectrometry yielding 51 HMO structures and isomers. Their peak areas were integrated and normalized to determine relative abundances. Univariate and multivariate analysis showed associations between relative HMO abundance and donors’ secretor status and specific infant anthropometric variables, but not with maternal pre-pregnancy BMI. This study does not support the hypothesis that maternal overweight influences the HMO profile and highlights the importance of reporting results despite absence of significant correlations.

## 1 Introduction

Obesity has many health and economic implications for the healthcare system and is therefore a major public health concern (1). Obesity has been associated with higher risks of developing type 2 diabetes (2), cardiovascular diseases (3), higher morbidity, and mortality (2–4). Elucidating the onset and development of obesity is important to advance preventative strategies, especially given the increasing prevalence of obesity across countries (5). While human milk has been recognized for its crucial role in optimal infant growth and development, maternal obesity appears to be a significant factor for childhood obesity (6). Formula feeding has also been associated with a higher risk of obesity later in childhood (7, 8), indicating that nutrition in early life is important for healthy growth in childhood. Human milk contains macronutrients, micronutrients, and bioactive compounds (9). Additionally, microbes including bacterial, viral, fungal, and archaeal components, as well as other compounds, some of which are important for the establishment and maintenance of the infant gut microbiome (10, 11).

An important fraction of human milk is composed of human milk oligosaccharides (HMOs), which are indigestible by the infant but serve as nutrients for select bacterial strains in the infant gut (9, 12, 13). In human colostrum, HMOs can reach a concentration of up to 20 g/L, similar to the protein content, and gradually decline to 8.6–16.8 g/L as the milk transitions to mature milk (14, 15). Furthermore, HMOs acts as antiviral (16) and immune-modulating agents (17). Research studies have presented convincing evidence that infants born to obese mothers exhibit a distinct gut microbiome composition compared to those born to normal weight mothers (18–20). By 1 month of age, infants born to an obese mother displayed a more diverse and more mature microbiome, which was related to elevated infant adiposity at 12 months (18). The indication that maternal obesity shifts the infant gut microbiota establishment could potentially be mediated through alterations in HMO composition and abundance.

The development of obesity is a complex process and is composed of many interplaying factors (21), one of which could be mediated through milk and the associated changes in infant gut microbiota composition. A sizeable body of research has recently emerged, focusing on associations between maternal BMI, HMO content, and infant growth parameters. Some studies report significant correlations between specific HMOs and maternal BMI, however, the results present conflicting outcomes as the significant correlations are not detected in all studies (22, 23). Furthermore, how specific HMOs are correlated to maternal BMI is also varying (22–33). Thus, the aim of this study was to investigate the relationship between HMO content and maternal pre-pregnancy BMI in otherwise healthy women from a Danish cohort, and additionally to infant anthropometrics. A review of recent related publications was conducted to contextualize the obtained results.

## 2 Materials and methods

### 2.1 Participants and sample collection

Participants were recruited from 2019 to 2020 as part of the MaInHealth cohort, established in Aarhus, Denmark, registered at ClinicalTrials.gov (identification number: NTC05111990) and approved by the Central Denmark Regional Committees of Health Research Ethics (approval reference: 1–10–72-296-18). All participants provided informed consent in accordance with the Declaration of Helsinki II. Participants were considered eligible if they expected to breastfeed for the first 4–6 months, had a BMI above 18.5 kg/m^2^, and anticipated delivering a singleton. Participants were excluded if the mother had a planned C-section, the infant was born pre-term (gestational age < 37 weeks), infant formula was utilized more than four times a week, or if the infant did not weigh between 2,500 g and 5,000 g at birth. Maternal and infant anthropometrics including maternal age, BMI, number of siblings, formula use, and infant weight and height recorded at 1 month postpartum were collected by self-reported questionnaires. Characteristics related to the birth including birth mode, birth weight, birth height, and gestational age were obtained from the electronic e-journal. A detailed description of the MaInHealth cohort including sample and information collection, as well as inclusion and exclusion criteria was reported in an earlier publication (34). Z-score calculations were performed in R statistical software using the addWGSR package relying on the World Health Organization growth reference z-scores.

Participants were given instructions on human milk collection, which involved manually expressing human milk around midday, at least 2 h after the last breastfeeding, while avoiding the first few drops. The milk was collected at 3 months postpartum in the participants’ own home in a 40 mL sterile container (Corning, Fisher Scientific) and kept in their own freezer at −18°C until the sample was collected and transported on dry ice within 14 days for storage at −70°C (34).

### 2.2 Sample preparation

Two hundred microliters of human milk were mixed with an equal volume of nanopure water and centrifuged at 4000 x *g* at 4°C for 30 min. 400 μL from the bottom aqueous layer was transferred to new tubes and the upper lipid layer was discarded. Four equivalent volumes of a 2:1 (v/v) mixture of CHCl_3_ and MeOH were added to the tube and vortexed. The samples were then centrifuged at 4000 x *g* at 4°C for 30 min. The MeOH layer, containing the oligosaccharides, was transferred to new tubes without disrupting the underneath layer containing proteins and lipids. Two equivalents of cold ethanol were added and placed at −20°C overnight. Samples were then centrifuged at 4000 x *g* at 4°C for 30 min to precipitate the proteins. Supernatants were transferred to new tubes and dried in a SpeedVac (MiVac Quattro, Genevac Ltd., Ipswitch, Suffolk, United Kingdom). The HMOs were further purified from the samples using solid-phase extraction (SPE), first with C18 (Bond Elute 50 mg, 1 mL, Agilent Technologies, Santa Clara, CA, United States) and then with graphitic carbon in a 96-well plate format. After each step of loading liquids, the SPE plate was centrifuged for 1 min at 20°C at 1000 rpm (for C18) or 800 rpm (for graphitic carbon) to assist the liquid flow through, unless otherwise stated. For the C18 extraction, wells in a 96-well plate were conditioned by adding 200 μL of 100% acetonitrile to each well and repeating this step once. This was followed by adding 200 μL of nanopure water twice. The dried samples were reconstituted in 200 μL of nanopure water and further diluted with nanopore water at a ratio of 1:9 (v/v). Subsequently, 80 μL of the 10-fold diluted samples were loaded into the C18 wells. Oligosaccharides were eluted over three rounds, each with 200 μL of nanopure water. The eluates were further purified with a self-packed graphitic carbon plate (35), with each well containing approximately 10 mg graphitic carbon sorbent (Supelclean ENVI-Carb SPE bulk packing, Millipore, Sigma, St. Louis, MO, USA), prepared essentially according to (35). Graphitic carbon wells were conditioned with two rounds of 200 μL of 80% acetonitrile and 0.1% trifluoroacetic acid in water (v/v/v), then with two rounds of 200 μL of nanopure water. Samples were loaded onto the conditioned wells. The graphitic carbon wells were washed with three rounds of 400 μL nanopure water. Oligosaccharides were collected over three rounds of elution, each with 200 μL of 40% acetonitrile and 0.1% trifluoroacetic acid in water (v/v/v), spinning the plate at 500 rpm for 1 min. The eluates were dried in a SpeedVac (MiVac Quattro).

The dried samples were reconstituted in 80 μL nanopure water. A pool of select samples was prepared and analyzed alongside reconstituted individual samples and used for HMO identification and relative quantification, respectively. For the pooled set, six representative HMO samples, matched by secretor status and BMI-group, were diluted with nanopure water to achieve an HMO concentration that was 100-fold diluted from their concentrations in the initial human milk. For individual samples, the reconstituted samples were diluted with nanopure water and spiked with an internal standard, xylosyl-cellobiose (Megazyme, Lansing, MI, United States), yielding a sample with HMO diluted 100 times from the initial human milk and containing 0.2 ng/μL xylosyl-cellobiose. The diluted pooled and individual samples were then filtered using 0.2 μm polyethersulfone 96-well filter plates (AcroPrep Advance, Supor, Pall, United States) before injection into the nano-LC-chip-QToF MS.

### 2.3 Mass spectrometry analysis

Mass spectrometry analysis was performed using an Agilent 6,520 accurate-mass Quadrupole-Time-of-Flight (Q-ToF) Liquid Chromatography Mass Spectrometer (LC–MS) with a microfluidic nano-electrospray chip (PGC-Chip II, G4240-64010, Agilent Technologies, Santa Clara, California). The chip contains an enrichment column and an analytical column, both packed with graphitized carbon, which is optimal for oligosaccharide isomeric separation. Two microliters of samples were delivered to the enrichment column by the capillary pump using 3% acetonitrile and 0.1% formic acid in nanopure water (v/v/v) (solvent A) at a flow rate of 4 μL/min. The binary gradient used for LC consisted of solvent A and solvent B (10% nanopure water and 0.1% formic acid in acetonitrile (v/v/v)). The column was initially equilibrated with solvent A at a 0.3 μL/min flow rate. The gradient was set to last 60 min and consisted of 0% B for 2.5 min, increasing from 0 to 16% B over 17.5 min, 16 to 44% B over the next 10 min, 44 to 100% B over the following 5 min, and maintained at 100% B for 10 min, followed by a rapid switch from 100 to 0% B in 0.01 min, and finally, held to 0% B for 14.99 min. The pooled sample was analyzed using tandem mass spectrometry (MS/MS) to obtain reliable fragmentation spectra for all major HMO peaks to create a library to be used for oligosaccharide identification in this dataset. The individual samples were then analyzed using MS profiling obtaining the retention time and accurate mass. Data were acquired in the positive ionization mode, with a mass-to-charge (*m*/*z*) range of 450–2,500 for MS and a range of 100–2,500 range for MS/MS. The electrospray capillary voltage was 1900–1970 V. The drying gas was at 350°C with a flow rate of 5 L/min. The acquisition rate was 1.0 spectra/s for both MS and MS/MS modes. In tandem MS analysis, automated precursor selection was employed based on abundance, with up to 8 MS/MS per cycle. The precursor isolation window was “medium (4 amu).” Fragmentation energy was set at 1.3 V/100 × (*m*/*z*) with an offset of −3.5 V. Internal continuous calibration was performed using the reference masses *m*/*z* 922.009 (Hexakis(1H, 1H, 3H-tetrafluoropropoxy)phosphazine) and 1221.991 (Hexakis(1H, 1H, 4H-hexafluorobutyloxy)phosphazine) (Agilent Technologies). Furthermore, an injection of a pure 2’FL standard was performed under the same MS settings for validation of secretor status determination.

### 2.4 Data analysis

Data from the pooled sample consisting of six human milk samples was used to generate an in-house HMO library with the software GlycoNote followed by manual inspection of all tandem MS/MS fragments (36). Only known HMO structures were included in the library (37), which consisted of 61 HMO structures with their corresponding monoisotopic mass and retention time. HMO peak area integration for the whole dataset was performed using Profinder B.08.00 (Agilent Technologies). The relative abundance of each HMO in every sample was obtained by normalizing their peak area to the peak area of the internal standard, xylosyl-cellobiose. Determination of secretor status was performed by visual inspection of the 2’FL peak in the human milk samples and comparison with the injection of the 2’FL standard.

Principal component analysis (PCA) was performed in Simca 17 (MKS Data Analytics Solutions, Umea, Sweden). Univariate analyses were performed in R statistical software (4.3.2). Statistically significant differences between maternal and infant characteristics according to maternal pre-pregnancy BMI-group were determined using analysis of variance (ANOVA) for normally distributed data or Kruskal Wallis for non-normally distributed data for continuous variables. Normality was tested with Shapiro–Wilk test. Post-hoc tests were performed with Wilcoxon signed-rank test and Tukey’s post-hoc test for Kruskal Wallis and ANOVA, respectively. Chi^2^ *post hoc* test was used for categorical data. After inspection of histograms representing the HMOs, data was log-transformed to obtain a near normal distribution. Relative abundances presenting zero were imputed with the minimum value divided by two. Linear regression was performed on the log-transformed data to investigate correlation between HMO relative abundances and infant and maternal characteristics including maternal age, secretor status, BMI, BMI-group, cesarean-section, birth weight, gestational age, infant sex, number of siblings and z-scores as listed in Table 1. Linear regression was also performed on data stratified according to secretor status as this variable is associated to HMO relative abundances as described in literature and identified in this study. Benjamini Hochberg was used as correction for multiple testing. Artificial intelligence assisted in the production of the R code and proof reading (38).

**Table 1:** Participant characteristics according to maternal pre-pregnancy BMI-group.

	NW (32)	OW (34)	OB (24)	*p*-value
Maternal characteristics				
Maternal age (years)	30.44 ± 4.49	31.74 ± 3.70	31.67 ± 4.69	*NS*
BMI (kg/m^2^)	22.22 ± 1.85^a^	26.83 ± 1.41^b^	34.45 ± 4.30^c^	*0.000*
Cesarean-section	3	1	1	*NS*
Infant characteristics				
Birth weight (kg)	3.65 ± 0.49	3.47 ± 1	3.84 ± 0.48	*NS*
Gestational age (days)	284.56 ± 6.38	278.97 ± 8.41	284.33 ± 7.23	*NS*
Infant sex (*n* female)	18	22	12	*NS*
Number of siblings (0/1/2/3)	(14/14/4/0)	(13/15/5/1)	(9/11/4/0)	*NS*
z-score birth	−0.08 ± 1.02	0.39 ± 0.98	0.34 ± 0.86	*NS*
HAZ (6 mo)	0.43 ± 1.35	0.42 ± 1.2	0.76 ± 1.77	*NS*
HAZ (1 y)	0.31 ± 1.52	0.29 ± 1.65	0.79 ± 2.04	*NS*
WAZ (6 mo)	0.32 ± 1.17	0.18 ± 0.95	0.73 ± 1.29	*NS*
WAZ (1 y)	0.33 ± 1.03	0.18 ± 2.03	0.72 ± 1.27	*NS*
WHZ (6 mo)	0.22 ± 1.19	0.03 ± 1.12	0.58 ± 1.32	*NS*
WHZ (1 y)	0.32 ± 1.18	0.12 ± 2.55	0.57 ± 1.14	*NS*

Continuous data presented as means ± standard deviation. Categorical data are presented as numbers included in each category. Small letters denote statistically significant differences between BMI-groups at a 0.05 significance level. NW, Normal weight; OW, overweight; OB, Obese; HAZ, height for age z-score; WAZ, weight for age z-score; WHZ, weight for height z-score; mo, months; y, year; NS, not significant.

### 2.5 Literature search

The PubMed database was searched using the query “human AND milk AND (oligosaccharide OR oligosaccharides) AND (obesity OR BMI OR infant)” displaying only papers published in English and published between 2017 and 2023, in total yielding 954 papers. Only original research works were being examined. Papers including measures of maternal BMI/obesity and reporting the results of the association were included in the review, in total consisting of 21 papers.

## 3 Results

Participant characteristics are shown in Table 1. Statistically significant differences between the BMI-groups were observed for BMI, as per design (34).

### 3.1 HMO characterization

In total 51 HMO structures, including isomers, were detected across all 90 samples. The 51 structures and their retention times are available in Supplementary Table S1. The relative abundances normalized to the internal standard xylosyl-cellobiose of the distinct structures are presented in Table 2. In addition, the HMOs were divided in four groups summing the total relative abundance for the main classes: fucosylated, sialyated, fucosylated and sialyated, and neutral HMOs. For 11 structures, including DF-LNH, F-LNH, F-LNO, FS-LNH, GL, LNFP, LNH + LNnH, LNO, LNT + LNnT, LST, and S-LNH (see legend to Table 2 for abbreviations) several isomers were detected. However, it was not possible to determine the exact structure, so they were denoted with “isomer” and a number was included after the name to distinguish them (Table 2).

**Table 2:** Average HMO relative abundance for the 51 structures identified in the 90 samples and further divided by BMI-group within the total sample set for secretors, and non-secretors.

	Mean ± standard deviation of relative HMO abundance								
	Total (90)			Secretor (71)			Non-secretor (19)		
Structure/isomer	NW (32)	OW (34)	OB (24)	NW (25)	OW (26)	OB (20)	NW (7)	OW (8)	OB (4)
2’FL	0.81 ± 0.51	0.86 ± 0.53	0.81 ± 0.45	1.02 ± 0.36	1.11 ± 0.32	0.96 ± 0.33	0.06 ± 0.03	0.06 ± 0.03	0.07 ± 0.04
3-FL	0.00 ± 0.00	0.00 ± 0.00	0.00 ± 0.00	0.00 ± 0.00	0.00 ± 0.00	0.00 ± 0.00	0.01 ± 0.00	0.00 ± 0.00	0.01 ± 0.00
3’SL	0.25 ± 0.16	0.24 ± 0.11	0.22 ± 0.13	0.24 ± 0.14	0.23 ± 0.11	0.22 ± 0.14	0.3 ± 0.21	0.27 ± 0.08	0.24 ± 0.09
6’SL	0.2 ± 0.16	0.3 ± 0.37	0.21 ± 0.19	0.2 ± 0.15	0.22 ± 0.22	0.19 ± 0.16	0.2 ± 0.18	0.55 ± 0.63	0.32 ± 0.3
3-SLN	0.00 ± 0.00	0.01 ± 0.01	0.01 ± 0.00	0.01 ± 0.00	0.01 ± 0.00	0.01 ± 0.00	0.00 ± 0.00	0.01 ± 0.01	0.00 ± 0.01
6-SLN	0.04 ± 0.04	0.05 ± 0.06	0.05 ± 0.07	0.04 ± 0.04	0.05 ± 0.03	0.05 ± 0.07	0.05 ± 0.04	0.08 ± 0.12	0.04 ± 0.01
DF-LND	0.02 ± 0.02	0.01 ± 0.01	0.02 ± 0.02	0.01 ± 0.02	0.01 ± 0.01	0.02 ± 0.02	0.02 ± 0.01	0.01 ± 0.01	0.01 ± 0.01
DF-LNH isomer 1	0.05 ± 0.05	0.05 ± 0.07	0.05 ± 0.07	0.04 ± 0.04	0.02 ± 0.02	0.04 ± 0.04	0.09 ± 0.05	0.13 ± 0.1	0.1 ± 0.15
DF-LNH isomer 2	0.09 ± 0.10	0.08 ± 0.11	0.09 ± 0.12	0.05 ± 0.06	0.03 ± 0.04	0.07 ± 0.07	0.24 ± 0.07	0.23 ± 0.13	0.19 ± 0.26
DF-LNH isomer 3	0.02 ± 0.02	0.02 ± 0.02	0.02 ± 0.02	0.03 ± 0.02	0.03 ± 0.02	0.03 ± 0.02	0.00 ± 0.00	0.00 ± 0.00	0.00 ± 0.00
DF-LNH isomer 4	0.01 ± 0.01	0.01 ± 0.01	0.01 ± 0.01	0.01 ± 0.01	0.01 ± 0.01	0.01 ± 0.01	0.00 ± 0.00	0.00 ± 0.00	0.00 ± 0.00
DF-LNO	0.02 ± 0.01	0.02 ± 0.02	0.02 ± 0.01	0.02 ± 0.01	0.02 ± 0.01	0.02 ± 0.01	0.02 ± 0.01	0.04 ± 0.04	0.02 ± 0.01
F-LND	0.02 ± 0.02	0.02 ± 0.02	0.03 ± 0.02	0.02 ± 0.02	0.02 ± 0.01	0.03 ± 0.02	0.02 ± 0.01	0.03 ± 0.03	0.02 ± 0.01
F-LNH isomer 1	0.04 ± 0.03	0.04 ± 0.05	0.05 ± 0.05	0.04 ± 0.03	0.04 ± 0.05	0.05 ± 0.04	0.05 ± 0.03	0.06 ± 0.04	0.06 ± 0.08
F-LNH isomer 2	0.54 ± 0.37	0.45 ± 0.33	0.51 ± 0.35	0.42 ± 0.31	0.35 ± 0.25	0.47 ± 0.32	0.97 ± 0.22	0.78 ± 0.38	0.72 ± 0.49
F-LNH isomer 3	0.5 ± 0.3	0.42 ± 0.28	0.5 ± 0.26	0.43 ± 0.28	0.36 ± 0.22	0.49 ± 0.25	0.76 ± 0.2	0.64 ± 0.36	0.55 ± 0.33
F-LNH isomer 4	0.33 ± 0.15	0.32 ± 0.15	0.36 ± 0.19	0.34 ± 0.17	0.33 ± 0.16	0.39 ± 0.19	0.32 ± 0.08	0.27 ± 0.14	0.22 ± 0.16
F-LNH isomer 5	0.23 ± 0.13	0.27 ± 0.14	0.27 ± 0.19	0.26 ± 0.13	0.3 ± 0.13	0.3 ± 0.19	0.14 ± 0.07	0.18 ± 0.11	0.12 ± 0.1
F-LNH isomer 6	0.24 ± 0.13	0.25 ± 0.13	0.28 ± 0.18	0.25 ± 0.14	0.28 ± 0.13	0.31 ± 0.19	0.2 ± 0.07	0.17 ± 0.1	0.15 ± 0.03
F-LNO isomer 1	0.06 ± 0.04	0.06 ± 0.06	0.07 ± 0.04	0.05 ± 0.04	0.05 ± 0.03	0.07 ± 0.04	0.09 ± 0.04	0.11 ± 0.1	0.07 ± 0.06
F-LNO isomer 2	0.11 ± 0.06	0.11 ± 0.08	0.12 ± 0.06	0.1 ± 0.07	0.09 ± 0.05	0.12 ± 0.06	0.13 ± 0.03	0.18 ± 0.12	0.11 ± 0.08
F-LNO isomer 3	0.04 ± 0.02	0.04 ± 0.03	0.05 ± 0.04	0.04 ± 0.02	0.05 ± 0.02	0.06 ± 0.04	0.02 ± 0.01	0.02 ± 0.01	0.02 ± 0.02
F-SL	0.02 ± 0.01	0.01 ± 0.01	0.02 ± 0.01	0.02 ± 0.01	0.01 ± 0.01	0.02 ± 0.01	0.01 ± 0.01	0.01 ± 0.00	0.01 ± 0.01
FS-LNH isomer 1	0.19 ± 0.13	0.21 ± 0.19	0.2 ± 0.13	0.18 ± 0.13	0.18 ± 0.15	0.2 ± 0.11	0.24 ± 0.16	0.32 ± 0.26	0.22 ± 0.24
FS-LNH isomer 2	0.11 ± 0.06	0.13 ± 0.07	0.12 ± 0.06	0.1 ± 0.06	0.13 ± 0.07	0.13 ± 0.06	0.12 ± 0.07	0.13 ± 0.07	0.12 ± 0.06
FS-LNH isomer 3	0.01 ± 0.01	0.01 ± 0.01	0.02 ± 0.03	0.02 ± 0.01	0.02 ± 0.01	0.03 ± 0.04	0.01 ± 0.00	0.01 ± 0.00	0.00 ± 0.00
FS-LNO	0.01 ± 0.01	0.01 ± 0.01	0.01 ± 0.01	0.01 ± 0.01	0.01 ± 0.01	0.01 ± 0.01	0.01 ± 0.00	0.01 ± 0.01	0.01 ± 0.00
GL isomer 1	0.02 ± 0.01	0.02 ± 0.01	0.02 ± 0.01	0.02 ± 0.01	0.02 ± 0.01	0.02 ± 0.01	0.02 ± 0.01	0.02 ± 0.01	0.02 ± 0.01
GL isomer 2	0.03 ± 0.02	0.03 ± 0.03	0.02 ± 0.01	0.03 ± 0.03	0.03 ± 0.02	0.02 ± 0.01	0.03 ± 0.01	0.05 ± 0.06	0.02 ± 0.01
GL isomer 3	0.07 ± 0.05	0.08 ± 0.07	0.06 ± 0.03	0.07 ± 0.06	0.08 ± 0.05	0.06 ± 0.03	0.05 ± 0.02	0.1 ± 0.1	0.05 ± 0.04
LDFT	0.63 ± 0.43	0.64 ± 0.47	0.65 ± 0.41	0.8 ± 0.33	0.82 ± 0.37	0.77 ± 0.33	0.04 ± 0.02	0.04 ± 0.01	0.04 ± 0.02
LND	0.06 ± 0.05	0.06 ± 0.04	0.08 ± 0.07	0.05 ± 0.05	0.05 ± 0.03	0.08 ± 0.07	0.08 ± 0.06	0.08 ± 0.04	0.06 ± 0.02
LNFP I	1.69 ± 1.23	1.89 ± 1.53	1.75 ± 1.27	2.14 ± 1	2.47 ± 1.29	2.09 ± 1.11	0.09 ± 0.04	0.03 ± 0.03	0.05 ± 0.06
LNFP II	1.1 ± 0.69	0.96 ± 0.66	1.08 ± 0.79	0.82 ± 0.42	0.7 ± 0.37	0.88 ± 0.49	2.06 ± 0.58	1.78 ± 0.76	2.07 ± 1.32
LNFP isomer 1	1.14 ± 0.9	1.33 ± 1.14	1.2 ± 0.97	1.44 ± 0.78	1.73 ± 1	1.43 ± 0.9	0.05 ± 0.04	0.04 ± 0.03	0.05 ± 0.08
LNFP isomer 2	1.07 ± 0.48	1.08 ± 0.43	1.11 ± 0.54	0.96 ± 0.41	0.93 ± 0.31	0.94 ± 0.4	1.49 ± 0.5	1.56 ± 0.39	1.93 ± 0.3
LNFP isomer 3	0.02 ± 0.02	0.03 ± 0.02	0.03 ± 0.02	0.02 ± 0.02	0.02 ± 0.01	0.03 ± 0.02	0.03 ± 0.02	0.04 ± 0.03	0.03 ± 0.02
LNFP isomer 4	0.02 ± 0.01	0.02 ± 0.01	0.02 ± 0.01	0.02 ± 0.01	0.02 ± 0.01	0.02 ± 0.01	0.03 ± 0.02	0.03 ± 0.01	0.04 ± 0.02
LNH + LNnH isomer 1	2.07 ± 0.65	2.14 ± 0.59	2.24 ± 0.71	1.99 ± 0.61	2.15 ± 0.59	2.28 ± 0.77	2.34 ± 0.76	2.09 ± 0.61	2.04 ± 0.18
LNH + LNnH isomer 2	0.24 ± 0.1	0.25 ± 0.11	0.26 ± 0.12	0.23 ± 0.09	0.24 ± 0.11	0.26 ± 0.13	0.28 ± 0.11	0.3 ± 0.11	0.26 ± 0.1
LNH + LNnH isomer 3	0.12 ± 0.06	0.14 ± 0.07	0.13 ± 0.06	0.13 ± 0.07	0.14 ± 0.07	0.14 ± 0.06	0.11 ± 0.04	0.11 ± 0.07	0.09 ± 0.02
LNO-isomer 1	0.05 ± 0.04	0.06 ± 0.04	0.05 ± 0.03	0.04 ± 0.04	0.06 ± 0.04	0.05 ± 0.04	0.07 ± 0.04	0.06 ± 0.05	0.07 ± 0.03
LNO-isomer 2	0.33 ± 0.15	0.36 ± 0.17	0.37 ± 0.17	0.33 ± 0.15	0.36 ± 0.17	0.39 ± 0.18	0.34 ± 0.13	0.34 ± 0.15	0.3 ± 0.09
LNT + LNnT isomer 1	3.85 ± 1.37	3.84 ± 1.64	4.1 ± 1.64	3.55 ± 1.18	3.36 ± 1.35	3.7 ± 1.41	4.95 ± 1.54	5.43 ± 1.52	6.14 ± 1.26
LNT + LNnT isomer 2	5.99 ± 1.43	6.18 ± 1.96	6.36 ± 1.86	5.78 ± 1.33	5.91 ± 1.95	6.1 ± 1.93	6.77 ± 1.61	7.03 ± 1.86	7.62 ± 0.67
LNTri-II	0.17 ± 0.14	0.18 ± 0.11	0.16 ± 0.1	0.19 ± 0.15	0.19 ± 0.1	0.16 ± 0.09	0.09 ± 0.04	0.16 ± 0.14	0.17 ± 0.14
LST isomer 1	0.3 ± 0.15	0.37 ± 0.35	0.32 ± 0.19	0.3 ± 0.15	0.33 ± 0.13	0.33 ± 0.2	0.27 ± 0.19	0.52 ± 0.69	0.28 ± 0.11
LST isomer 2	0.29 ± 0.11	0.28 ± 0.09	0.27 ± 0.1	0.27 ± 0.09	0.26 ± 0.07	0.25 ± 0.08	0.36 ± 0.16	0.36 ± 0.11	0.4 ± 0.12
S-LNH isomer 1	0.81 ± 0.28	0.93 ± 0.37	0.9 ± 0.27	0.82 ± 0.3	0.91 ± 0.3	0.91 ± 0.28	0.8 ± 0.25	0.99 ± 0.58	0.82 ± 0.27
S-LNH isomer 2	0.03 ± 0.02	0.04 ± 0.05	0.04 ± 0.04	0.03 ± 0.02	0.03 ± 0.03	0.04 ± 0.05	0.03 ± 0.03	0.06 ± 0.08	0.04 ± 0.01
TF-LNH	0.03 ± 0.04	0.02 ± 0.02	0.03 ± 0.03	0.04 ± 0.04	0.03 ± 0.03	0.04 ± 0.03	0.00 ± 0.00	0.00 ± 0.00	0.00 ± 0.00
Total fucosylated	8.84 ± 2.47	9.03 ± 3.3	9.13 ± 2.63	9.37 ± 2.49	9.82 ± 3.24	9.63 ± 2.27	6.95 ± 1.14	6.45 ± 1.97	6.66 ± 3.26
Total sialyated	1.93 ± 0.66	2.22 ± 1.19	2.02 ± 0.72	1.9 ± 0.62	2.03 ± 0.7	2 ± 0.73	2.02 ± 0.84	2.85 ± 2.09	2.14 ± 0.78
Total fucosylated and sialyated	0.34 ± 0.19	0.38 ± 0.25	0.38 ± 0.2	0.33 ± 0.18	0.35 ± 0.21	0.38 ± 0.18	0.38 ± 0.2	0.47 ± 0.33	0.36 ± 0.31
Total neutral	12.99 ± 3.33	13.33 ± 4.26	13.85 ± 4.03	12.39 ± 3.11	12.59 ± 4.11	13.26 ± 4.08	15.14 ± 3.42	15.76 ± 4.04	16.83 ± 2.21

FL, fucosyllactose; SL, sialyllactose; SLN, sialyllactosamine; DF, difucosyl; LND, lacto-*N*-decaose; LNH, lacto-*N*-hexaose; LNO, lacto-*N*-octaose; F, fucose; FS, fucosyl sialyl; GL, galactosyllactose; LDFT, lacto difucotetraose; LNFP, lacto-*N*-fucopentaose; LNnH, lacto-*N*-neohexaose; LNT, lacto-*N*-tetraose; LNnT, lacto-*N*-neotetraose; LST, sialyllacto-*N*-tetraose; TF, trifucosyl.

### 3.2 Relationship between HMO relative abundances and maternal BMI

**Figure 1:**
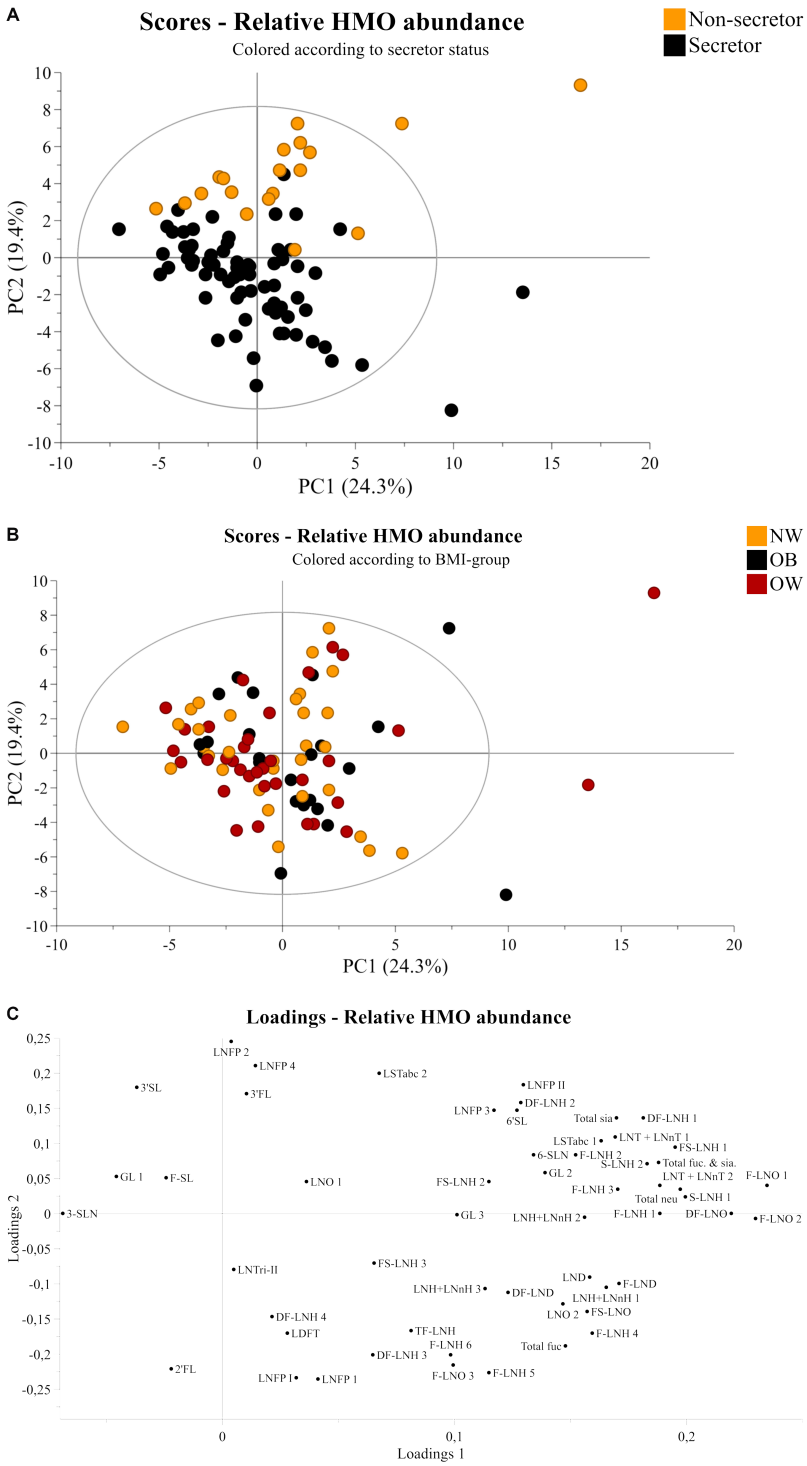
Principal component analysis of human milk samples (*n* = 90). Scores plot of all samples colored according to **(A)** secretor status or **(B)** BMI-group showing principal component one and two on the x-axis and y-axis, respectively. **(C)** Corresponding loadings plot showing all 51 HMO structures and isomers. NW, Normal weight; OB, Obese; OW, Overweight; FL, fucosyllactose, SL, sialyllactose; SLN, sialyllactosamine; DF, difucosyl; LND, lacto-*N*-decaose; LNH, lacto-*N*-hexaose; LNO, lacto-*N*-octaose; F, fucose; Fuc, Fucosylated; FS, fucosyl sialyl; GL, galactosyllactose; LDFT, lacto difucotetraose; LNFP, lacto-*N*-fucopentaose; LNnH, lacto-*N*-neohexaose; LNT, lacto-*N*-tetraose; LNnT, lacto-*N*-neotetraose; LST, sialyllacto-*N*-tetraose; neu, neutral; sia, sialyated; TF, trifucosyl.

*P*-values indicating statistical significance of HMO relative abundances between BMI-groups are not presented, as differences between BMI-groups or linear correlations to BMI values after analysis with linear regression were not found. This was also true when stratifying according to secretor status. Results from the linear regression analysis of the total samples set, secretors, and non-secretors are presented in the Supplementary Tables S2–S4, respectively. Further, a PCA was performed to determine potential patterns related to BMI-groups. However, as shown in Figure 1, no distinct separation of samples according to BMI-group was observed based on the HMOs measured in this study (Figure 1B).

### 3.3 HMO relative abundances and relation to secretor status and infant anthropometrics

Secretor status was determined based on the 2’FL abundance. A clear discrepancy was observed in terms of peaks corresponding (or not) to 2’FL in secretors and non-secretors (Supplementary Figure S1). Injection of the pure 2’FL standard showed that the 2’FL compound eluted at 11.5–12.5 min, and this was used as a reference to determine the retention time and peak area, complemented by the tandem MS/MS fragmentation data on the pooled samples (Supplementary Figure S2). Thus, secretor status was determined from inspection of spectra. In accordance with the expected distribution for secretors and non-secretors in the European population (39), 71 mothers were determined to be secretor positive and 19 were non-secretor. A PCA was performed based on relative abundances of all HMOs in the dataset. Scores plots of principal components 1 and 2, colored according to secretor status or BMI and the corresponding loadings plot can be viewed in Figure 1. The figure shows a clear division related to secretor status (Figure 1A). The loadings plot in Figure 1C shows that 2’FL, LNFP I and LDFT are positively correlated with secretors, while LNFP II, 3-FL, and LNFP isomer 2 are positively correlated with non-secretors.

The linear regression model showed statistically significant differences between secretors and non-secretors in terms of 2’FL, LDFT, LNFP II, DF-LNH isomer 2, LNFP I, LNFP isomer 1, LNFP isomer 2, DF-LNH isomer 3, DF-LNH isomer 1, F-LNH isomer 2, LNT + LNnT isomer 1, LST isomer 2, 3-FL, F-LNO isomer 3, total fucosylated, DF-LNH isomer 4, TF-LNH, F-LNH isomer 5, F-LNH isomer 3, LNFP isomer 4, F-LNO isomer 1, total neutral, listed from the lowest to highest *p*-value after Benjamini Hochberg correction (Supplementary Table S2). These results are in agreement with previously published literature (24, 28, 33).

When stratifying according to secretor status, two HMO structures were significantly associated to infant anthropometrics for the non-secretors (Supplementary Table S4). DF-LNH isomer 4 were positively correlated to two number of siblings, however, only one infant of a non-secretor mother had two siblings (Supplementary Table S4). Additionally, the weight for height z-score (WHZ) at 1 year was negatively correlated to the FS-LNH isomer 3 abundance (Supplementary Table S4).

## 4 Discussion

### 4.1 Variation of human milk oligosaccharides and relation to maternal body mass index in published literature

This study was designed to examine potential differences in HMO composition between BMI-groups among 90 healthy mothers at 3 months postpartum. Interestingly, no significant correlations between BMI and HMO relative abundances were identified. A total of 51 different HMOs were measured across all samples, representing the most abundant HMO species.

Numerous studies have examined the association between HMOs and maternal BMI. A summary of recent published findings, which includes conflicting results, is presented in Table 3. The table contains results regarding 13 of the most abundant HMOs in human milk including 2’FL, DFLNT, LNFP I, LNFP II, LNFP III, LNT, LNnT, 3-FL, 3’SL, 6’SL, DSLNT, LDFT, and LNH. Supplementary Table S5 contains results of all HMOs reported with significant associations in the presented papers.

**Table 3:** Summary of results from studies investigating human milk oligosaccharides (HMOs) and BMI/overweight/obesity and infant anthropometrics for 13 of the most abundant HMOs stratified according to secretor status.

Reference and included participants	Time of sample and data collection. HMO analysis method	HMO association to maternal BMI or overweight/obesity			HMO association to infant anthropometrics		
		*Secretor independent*	*Secretors*	*Non-secretors*	*Secretor independent*	*Secretors*	*Non-secretors*
Larsson et al. (22) 30 mother-infant dyads	Human milk: 5–6½ and 9 months Infant anthropometrics: 5–6½ and 9 months Analysis method: HPLC after fluorescent derivatization. Raffinose as internal standard After correction for multiple testing, results were not significant, thus raw *p*-values were reported.	BMI at 5 months postpartum: ↑ 2’FL ↓ 6’SL ppBMI: ↑ 2’FL				High weight gain infants: 5 months: ↓ LNnT 9 months: ↓ LNnT Infant growth at 5 months: HAZ: ↑ 3’SL ↓ LNnT BAZ: ↓ 6’SL Weight velocity from 0–5 months: ↑ 2’FL ↓ LNnT Fat mass index at 5 months: ↑ 2’FL ↓ LNnT	
Lagström et al. (28) 802 mother-infant dyads	Human milk: 3 months Infant anthropometrics: 3, 6, 8 months 1, 2, 3, 4, 5 years Analysis method: HPLC after fluorescent derivatization. Raffinose as internal standard No mention of correction for multiple testing	HMO concentration, ppBMI: ↓ LNnT	HMO concentration, ppBMI: ↓LNnT			WAZ 3–12 months: ↑2’FL, 3’FL, 3’SL ↓ LNnT HAZ 3–12 months: ↑ 2’FL ↓ LNnT HAZ 1–5 years: ↓ LNnT WAZ 1–5 years: ↑ 3’FL, 3’SL ↓ LNnT	WAZ 3–12 months: ↓ 6’SL
Saben et al. (23) 194 mother-infant dyads	Human milk: 2 months Infant anthropometrics: 2 and 6 months Analysis method: HPLC after fluorescent derivatization. Raffinose as internal standard. No mention of correction for multiple testing.	HMO concentration: ppBMI value, ↑ LNnT, 3’FL, 6’SL ↓ DFLNT, DSLNT HMO intake: ppBMI-group OB: ↓DFLNT, LNH	HMO concentration: ppBMI-group: OW: ↓ LNT, LNnT OB: ↑ LNnT, 3’FL, DFLNT ↓DSLNT, LNH	HMO concentration: ppBMI-group: OW: ↓ 3’SL	Fat mass 2–6 months: ↑ 3’FL, LNFP II, LNFP III, 6’SL, DSLNT, WLZ 2–6 months: ↑ LNT, 3’FL, LNFP II WAZ 2–6 months: ↑ 3’FL, LNFP II, 3’SL		
Cheema et al. (24) 60 mother-infant dyads	Human milk: 3 months Infant anthropometrics: 3 months Analysis method: HPLC after fluorescent derivatization. Raffinose as internal standard. No correction for multiple testing	Logarithm of BMI at 3 months postpartum: ↑ 2’FL, Log(LNH)			HMO concentration: LAZ: ↓ Log(LNnT) Fat mass, fat mass to fat-free mass ratio: ↓Log(LNFP III) HMO intake: Infant BMI: ↑ DFLNT WAZ: ↑3’FL	HMO concentration: Log(fat free mass): ↑3’SL Fat mass: ↑ DFLNT HMO intake: WAZ, log(fat-free mass): ↑Log(3’SL)	HMO concentration: WAZ: ↑ DFLNT HAZ: ↑ DFLNT HMO intake: WAZ, fat mass, fat index: ↓ 6’SL
Tonon et al. (32) 78 mother-infant dyads	Human milk: 17–76 days Infant anthropometrics: 17–76 days Analysis method: Liquid-chromatography mass spectrometry. No mention of correction for multiple testing		BMI-group at sampling time: OW: ↑ 2’FL ↓ 3’FL BMI-value at sampling time: ↑ 2’FL Maternal ppBMI: ↑2’FL			Weight gain: ↓ LNFP, LNDFH I, 6’SL	
Menzel et al. (30) 145 mother-infant dyads	Human milk: 3 months Infant anthropometrics: 6 months 1, 2, 3, 4, 5, 6, 7 years Analysis method: Nuclear magnetic resonance spectroscopy. No correction for multiple testing			ppBMI: ↓ LNnT	Height-SDS ↑ LNnT	Heigh-SDS: ↑ LNT Growth velocity: ↓ LNT, LNFP-I, 3’SL BMI-SDS: ↑3’FL ↓2’FL	Height-SDS: ↓ LNT Growth velocity: ↓ LNnT BMI-SDS: ↓ 3’SL, 6’SL, LNT
Wang et al. (33) 116 mother-infant dyads	Human milk: 1–5 days 8–14 days 1 month 6 months Infant anthropometrics: 1–5 days 8–14 days 1 month 6 months Analysis method: HPLC mass spectrometry. No correction for multiple testing		ppBMI: 1 month: ↑ 2’FL, LNH	ppBMI: 1 month: ↓ 3’FL 6 months: ↓ LNFP-II		Month 6 weight gain and human milk 6 months: ↑ 3’FL, LDFT, ↓ LNT&LNnT, LNFP-I, LNH Month 1 length gain and human milk 1–5 days: ↑ LNH Month 1 weight gain and human milk 1–5 days: ↓ LNFP-II	Month 6 weight gain and human milk 6 months: ↓ 3’FL Month 1 length gain and human milk 1–5 days: ↑ LNT&LNnT, Month 1 length gain and human milk 8–14 days: ↑ LNT&LNnT
Mainardi et al. (29) 107 mother-infant dyads	Human milk: 2-5 weeks 1.5 months 3 months Infant anthropometrics: 2–5 weeks 1.5, 3, 6, 9, 12, 18, 24 months Analysis method: Ultra HPLC with fluorescence detection. Benjamini–Hochsberg correction for multiple testing with a false discovery rate of 5%.	Average trajectories over time between HMOs and maternal BMI-groups: OB: ↑ LNH			Length velocity: ↓ 3’FL, DSLNT, LNFP-II, LNT		
Isganaitis et al. (27) 31 mother-infant dyads	Human milk: 1 and 6 months Infant anthropometrics: 1 and 6 months Analysis method: Liquid chromatography- gas chromatography – mass spectrometry. After correction for multiple testing, results were not significant, thus raw *p*-values were reported.	Maternal ppBMI, human milk 1 month: ↑ LNFP-II/III ↓ 2’FL, LNFP-I			No significant correlation between HMOs and infant anthropometrics		

Studies including HMOs and association to maternal BMI/obesity				
Reference and included participants	Time of sample and data collection. HMO analysis method	HMO association to BMI or overweight/obesity		
		*Secretor independent*	*Secretor*	*Non-secretor*
Fan et al. (25) 392 mother-infant dyads	Human milk: 1.5 months Analysis method: HPLC-Chip-Time-of-Flight mass spectrometry. Correction for multiple testing by Bonferroni	ppBMI-group: OW: ↑ 3’SL		ppBMI-group: OW: ↓ LNFP I + III OB: ↑ 3’FL ppBMI-value: ↓ 3’FL
Azad et al. (40) 427 mother-infant dyads	Human milk: 3–4 months Analysis method: HPLC after fluorescent derivatization. Raffinose as internal standard. Correction for multiple testing by FDR-adjustment.	ppBMI: OW: ↓ LNH OB: ↑ DFLNT *Not significant after FDR correction, which is also highlighted in paper.		
Samuel et al. (31) 290 mother-infant dyads	Human milk: 2 and 17 days 1, 2, 3, 4 months Analysis method: HPLC after fluorescent derivatization in combination with nuclear magnetic resonance spectroscopy. Correction for multiple testing by Benjamini–Hochberg.	ppBMI: OW/OB: 2 days: ↑ 3’SL, DSLNT ↓ LNnT 17 days: ↑ 6’SL 1 month: ↓ LNT 3 months: ↓ LNT *After correction for multiple testing only 3’SL remained significantly different		
Ferreira et al. (26) 101 mother-infant dyads	Human milk: 2–8 days 28–50 days 88–119 days Analysis method: HPLC after fluorescent derivatization. Raffinose as internal standard. No mention of correction for multiple testing	ppBMI 2–8 days: ↑ LNnT 88–119 days: ↓ LNnT		
McGuire et al. (42) 410 mother-infant dyads	Human milk: Between 2 weeks and 5 months Analysis method: HPLC after fluorescent derivatization. Raffinose as internal standard. No correction for multiple testing, however significance level at 0.01	BMI, time not specified: ↑ 2’FL, HMO-bound fucose ↓ LNnT, DSLNT		
Neville et al. (50) 74 mother-infant dyads	Human milk: At least 2 weeks postpartum Analysis method: HPLC after fluorescent derivatization. Raffinose as internal standard. Correction for multiple testing by Bonferroni.	Maternal BMI: ↑ 3’SL		

Studies obtaining no significant associations between HMOs and maternal BMI/obesity				
Reference and included participants	Time of sample and data collection. HMO analysis method	HMO association to BMI or overweight/obesity		
		*Secretor independent*	*Secretor*	*Non-secretor*
Siziba et al. (47) 970 mother-infant dyads	Human milk: 6 weeks 6 and 12 months Analysis method: Label-free targeted liquid chromatography mass spectrometry. Correction for multiple testing by Bonferroni.	NS	NS	NS
Berger et al. (43) 50 mother-infant dyads	Human milk: 1 and 6 months Analysis method: HPLC after fluorescent derivatization. Raffinose as internal standard. No mention of correction for multiple testing.	NS	NS	NS
Biddulph et al. (44) 101 mother-infant dyads	Human milk: 3–4 months Analysis method: Liquid chromatography mass spectrometry. Correction for multiple testing by either Benjamini–Hochberg or Bonferonni.	NS	NS	NS
Ren et al. (46) 481 mother-infant dyads	Human milk: 0–6 days, 7–14 days, 15–340 days Analysis method: UPLC multi-reaction monitoring mass spectrometry. Correction for multiple testing by Tukey–Kramer.	NS	NS	NS
Zhang et al. (48) 203 mother-infant dyads	Human milk: 15–180 days Analysis method: UPLC multi-reaction monitoring mass spectrometry. Correction for multiple testing by either Benjamini–Hochberg.	NS	NS	NS
Pell et al. (45) 192 mother-infant dyads	Human milk: ~93 days Analysis method: HPLC after fluorescent derivatization. Raffinose as internal standard. Correction for multiple testing by Holm method.	NS	NS	NS

↑ denote a significant positive correlation, ↓ denote a significant negative correlation between the given characteristic and the listed HMO. 2’FL, 2′-fucosyllactose; 3’FL, 3’fucsyllactose; 3’SL, 3′-sialyllactose; 6’SL, 6′-sialyllactose; BAZ, BMI-for-age Z-scores; DFLNT, difucosyllacto-N-tetrose; DSLNT, disailyllacto-N-tetraose; HAZ, Height-for-Age Z-scores; HMO, human milk oligosaccharides; LDFT, lactodifucotetraose; LNFP, Lacto-N-fucopentaose; LNnT, lacto-N-neotetraose; LNH, lacto-N-haxaose; LNT, lacto-N-tetraose; OB, obese; OW, overweight; ppBMI, pre-pregnancy BMI; SDS, standard deviation score; UPLC, ultra-performance liquid chromatography; WAZ, Weight-for-age Z-score; WLZ, weight-for-length Z-score.

Notably, some investigators (22) discovered a positive correlation between pre-pregnancy BMI and 2’FL in milk samples collected at 5 months postpartum, across both secretors and non-secretors. Several other studies also demonstrated that higher concentrations of 2’FL in milk were associated with higher maternal BMI (24, 27, 28, 33). For some studies, the correlation between 2’FL and BMI were only seen for secretors, aligning with non-secretors producing very low amounts of 2’FL (28, 32). A greater number of studies examined the same correlation yet did not obtain statistically significant values for 2’FL and BMI (23, 25, 26, 29–31, 40). One study (23) showed a trend toward a positive correlation, which was only observed for overweight mothers, but not for obese mothers, aligning with results from others (32).

Besides the work focusing on 2’FL, another extensively studied HMO is LNnT. Along with 2’FL, LNnT are nowadays supplemented to infant formula, and yet yielded inconsistent results (41). Lower concentrations of LNnT have been reported in overweight mothers (73.3 ± 5.9 μg/mL), compared to normal weight mothers (100.5 ± 7.9 μg/mL) (23); whereas obese mothers displayed the highest concentration (154.7 ± 11.5 μg/mL). The presented significant differences between BMI-groups were only from secretors, values in non-secretors did not present significant differences (23). An inverse correlation between LNnT and BMI have also been reported, however, the highest BMI-value in that study was 25.8 kg/m^2^ in the secretor group (28). Accordingly, it remains unclear if this correlation is also valid in obese mothers defined by a BMI above 30 kg/m^2^. To be able to present results from obese mothers, the present study includes participants with BMI up to 46 kg/m^2^ that are thereby considered obese. Oppositely, a significant negative correlation between BMI and LNnT were found in non-secretors, but not in secretors (30). Other studies did not establish any correlation between LNnT and BMI (22, 24, 25, 29, 32, 33, 40).

Besides 2’FL, other fucsosylated HMOs were also investigated and presented inconsistent associations. The ones presented in Table 3 include 3’FL, DFLNT, and LNFP II/III. For example, concentrations of 3’FL were positively correlated to pre-pregnancy BMI value independent of secretor status in one study (23), while it was negatively correlated with the OW-group for secretors in another study (32).

Regarding major acidic oligosaccharides such as 3’SL, conflicting results were also reported. One study documented significantly lower 3’SL levels in the milk of overweight mothers compared to the obese mothers among non-secretors, with a similar trend for secretors (23). Oppositely, a more recent publication reported higher levels of 3’SL in milk from overweight mothers compared to obese mothers, irrespective of secretor status (25). In addition to 3’SL, 6’SL and DSLNT have also been investigated and conflicting results were also presented for these. For 6’SL a negative correlation to BMI has been reported in one study (22), while two studies reported a positive correlation all reported independent of secretor status (23, 31). Similarly, conflicting results were also observed for DSLNT (Table 3) (23, 31, 42).

Conflicting results were also observed for the neutral HMOs LNH and LNT. For example, the overall trajectory of LNH was positively correlated to the OB-group (29), while intake of LNH was negatively correlated to the OB-group in another study (23). A third study presented a negative correlation between LNH concentration and the OW-group (40). The mentioned results for LNH were all independent of secretor status (Table 3). Additionally, several studies represent results in accordance with ours, and do not observe correlations between any HMOs and maternal BMI (43–48).

The lack of consistency across studies may stem from variation in analysis technique, stratification based on secretor status, sample sizes, and timing of sample collection. Additionally, a limitation presented in the different studies is the lack of correction for multiple testing or choosing to present raw *p*-values instead of corrected ones, which could potentially lead to presentation of false positive discoveries. Although, it is worth mentioning that some studies do report their results after correction for multiple testing (Table 3).

### 4.2 Human milk oligosaccharides and relation to infant growth parameters

In addition to BMI, infant growth parameters have also extensively been studied in relation to HMOs to elucidate the complex relationship between human milk composition and infant growth. Notably, our study found a significant negative correlation between a FS-LNH isomer and infant WHZ at 1 year. This association has to our knowledge, not been observed before. A summary of the published literature findings is available in Table 3. These investigations exhibit considerable variability due to differences in the timing of milk sampling, methods for assessing infant growth, duration of follow-up assessment, and the time point selected for the association. One study extended the anthropometrics measurements up to 7 years postpartum, while the collection of human milk was at 3 months postpartum (30). Several different associations for specific HMOs have been reported, also showing inconsistencies. For LNnT, a frequently analyzed HMO, some studies align with our results and find no correlation (23), whereas others correlated it inversely with infant growth measures (22, 24, 28), and yet some other studies correlated it positively with infant growth (30, 49). The inconsistencies among findings underscore the complexity of this relationship. This work underlines that it is crucial to exercise caution when relating HMO structures/abundances to infant growth metrics since many confounding factors come into play, especially when considering extended periods after birth. Some examples of such cases are given in Table 3, correlating HMOs from milk collected at 3 months and infant anthropometrics at 24 months (29), human milk samples collected at 3 months postpartum and the correlation on infant anthropometrics was extended to 1–5 years of age (28), or human milk collection at 3 months postpartum and infant anthropometrics up to 7 years of age (30). When investigating correlation on such extended time frames, discerning whether the correlation also implies causation between the particular HMO and infant growth becomes questionable, especially when taking into account the short duration of breastfeeding in relation to when measurements of infant anthropometrics were taken, such as at 5 or 7 years of age.

### 4.3 Human milk oligosaccharide abundances and secretor status

In total, 71 participants were phenotyped as secretors and 19 as non-secretors. This distribution of secretors/non-secretors is in agreement with the expected values for a European population (14). In alignment with existing published literature, we observed a high abundance of 2’FL for secretors, yet our data showed some baseline peaks matching to the retention time of 2’FL for the non-secretors as well, albeit to a much lower level (Supplementary Figure S1). A similar result was observed in an earlier paper (39) in which the concept of weak secretors is extensively discussed.

### 4.4 Strengths and limitations

The present study was designed to investigate correlations between BMI and HMO abundances in human milk. It presents a sample size of 90 participants with BMI-values ranging from 18.5 to 46 kg/m^2^. Further, we detected 51 HMO structures across all samples using chip Q-ToF MS for high-throughput sensitive analysis. To ensure data reliability, an internal standard was added to each sample to account for potential variation in the ionization efficiency in LC–MS analysis over the many days required to run this sample set, assuring high quality of the relative HMO abundances measured. Importantly, the study included both secretor and non-secretor individuals, allowing for stratification based on secretor status, providing insights into potential associations across different phenotypes. Our analytical approach involved linear regression with correction for multiple testing given the high number of measured variables, thus reducing the risk of false positives. Nevertheless, we acknowledge some limitations in our study. Although adjusting the *p*-values reduces the risk of reporting false positives, it increases the risk of reporting false negatives, particularly considering the sample size of our study. Analyzing multiple dependent and independent variables increases the risk of reporting false positives. One key objective of this study was to minimize the occurrence of these errors. It is important to note that strong correlations, such as those observed between HMOs and secretor status, remain statistically significant suggesting that the findings are robust. We did not measure absolute concentrations or infant intake of the HMOs as we aimed to monitor a high number of structures for which standards are not yet available. Additionally, the complexity of HMO structures posed challenges in discriminating between isomers for which, again, pure standards are not yet fully commercially available. Due to those factors, only a select number of specific structures were identified by comparing their retention times with pure standards. Although identifying other isomers could be achieved through alternative and time-consuming approaches, it was not the primary objective of this study. We also observed a relatively low abundance of 3-FL values due to its weak binding to graphitic carbon, the chromatographic sorbent used in this study. Nonetheless, 3-FL abundances are still significantly higher in non-secretors compared to secretors, which has also been observed before using other analytical methods (39).

Despite the limitations, our study provides useful knowledge in understanding the complexity of human milk and further highlights the importance of reporting results even when significant correlations are not observed. Further research should focus on how the HMOs affect the infant gut microbiota and their implications on infant growth.

## 5 Conclusion

In conclusion, the conflicting findings in the literature regarding the relationship between HMOs and maternal BMI/infant anthropometric measurements suggest that potential correlations may be weak and of limited biological relevance. Although the present study does not fully resolve the issues raised regarding the correlation between maternal BMI, HMO content, and infant growth parameters, it calls for the scientific community to pay attention as industry are actively looking at published data to establish the appropriate amount of recombinant HMO to be included into their infant formula products. Further research with larger sample sizes, standardized methodologies, rigorous statistical analysis, and meta-analysis of published results are necessary to elucidate the interplay between maternal overweight, HMO composition, and infant growth.

## Data Availability

The raw data supporting the conclusions of this article will be made available by the authors, without undue reservation.

## Supplementary material

SUPPLEMENTARY TABLE S1 The total 51 HMO structures and their respective retention time.

SUPPLEMENTARY TABLE S2 Results from the linear regression analysis on the total sample set and HMOs. With the estimate, standard error, *p*-value and Benjamini Hochberg adjusted *p*-value for the different HMOs and the term investigated.

SUPPLEMENTARY TABLE S3 Results from the linear regression analysis from secretor mothers and HMOs. With the estimate, standard error, *p*-value and Benjamini Hochberg adjusted *p*-value for the different HMOs and the term investigated.

SUPPLEMENTARY TABLE S4 Results from the linear regression analysis from non-secretor mothers and HMOs. With the estimate, standard error, *p*-value and Benjamini Hochberg adjusted *p*-value for the different HMOs and the term investigated.
