# Supplementary material for: Human milk oligosaccharide profiles remain unaffected by maternal pre-pregnancy body mass index in an observational study
Source: Front Nutr. 2024 Oct 16;11:1455251. doi: 10.3389/fnut.2024.1455251 (PMC11523534; doi:10.3389/fnut.2024.1455251)
Supplement: Supplementary file 2 [file Image_1.pdf]

Supplementary Material

1 Supplementary Data

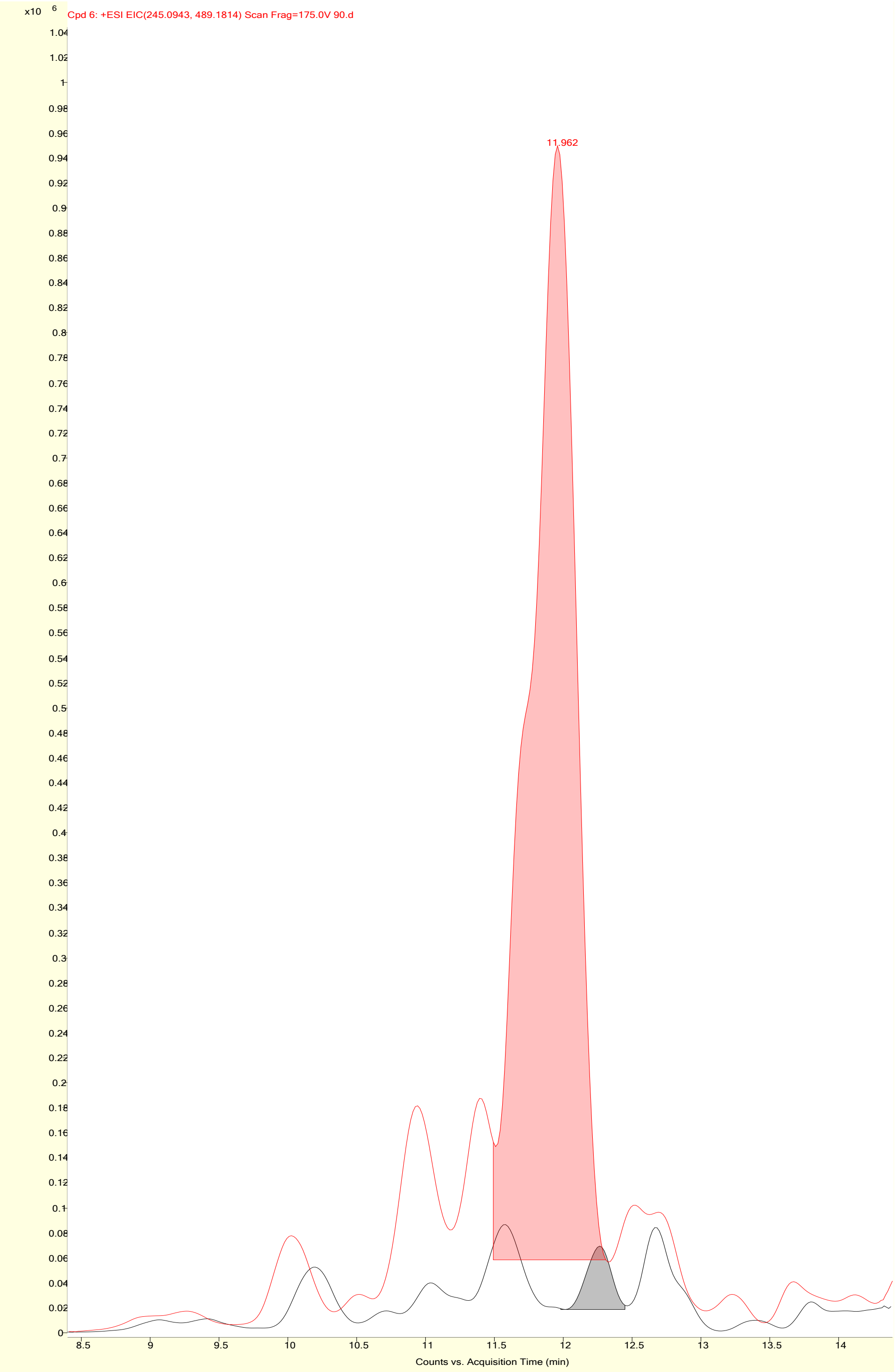

Supplementary Figure 1. Overlay of 2'FL spectra from a secretor sample (red) and a non-secretor sample (black) from Profinder B.08.00

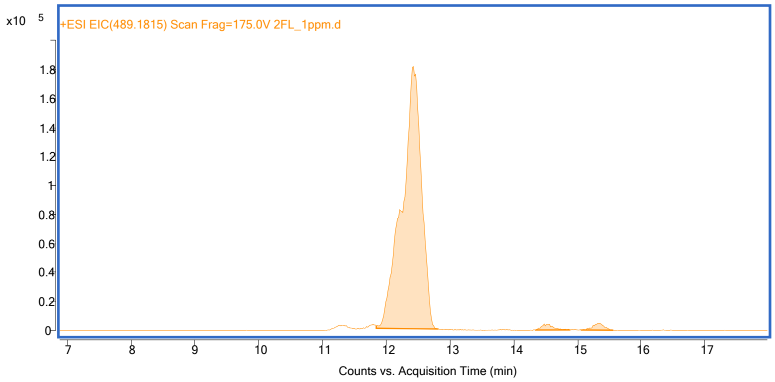

Supplementary Figure 2. Injection of 2'FL standard with a concentration of 1 µg/mL.
